# Manifestaciones orales de la COVID-19 y el rol del receptor ACE2: ¿qué se sabe hasta el momento?

**DOI:** 10.21142/2523-2754-1002-2022-108

**Published:** 2022-06-27

**Authors:** Ivo Luna-Mazzola

**Affiliations:** 1 Sociedad Científica de Estudiantes de Odontología, Universidad Nacional Mayor de San Marcos. Lima, Perú. ivo00lunamazzola@gmail.com Universidad Nacional Mayor de San Marcos Sociedad Científica de Estudiantes de Odontología Universidad Nacional Mayor de San Marcos Lima Peru ivo00lunamazzola@gmail.com

**Keywords:** infección por coronavirus, COVID-19, SARS-CoV-2, manifestaciones bucales, cavidad oral, coronavirus infections, COVID-19, SARS-CoV-2, oral manifestations, oral cavity

## Abstract

La COVID-19 es una enfermedad actualmente pandémica que preocupa a profesionales de todas las áreas de las ciencias de la salud. La literatura actual indica que ciertos componentes del sistema estomatognático como lengua y periodonto expresan los receptores ACE2, responsables de la unión del SARS-CoV-2 con la célula humana. Esta expresión repercute en un marcado compromiso organular y convierte a la cavidad oral no solo en una importante vía de ingreso viral, sino también en una región con posibilidad de diversas manifestaciones como la xerostomía, la halitosis, la alteración quimiosensorial, la coinfección oportunista y las lesiones de mucosa. El reporte de estas manifestaciones en etapas tempranas de la enfermedad da indicios de un posible carácter predictivo y puede ser de gran utilidad para la derivación diagnóstica. En tal sentido, es importante que el profesional odontólogo sepa reconocer esta sintomatología de presentación temprana, para así detectar presuntivamente pacientes con COVID-19 en quienes aún no se haya consolidado un compromiso sistémico significativo. Esto permitiría derivar a interconsulta médica con fines diagnósticos definitivos y, de estar en lo correcto, prevenir tanto contagios como el avance de la enfermedad en el paciente. Fundados en esta importante labor, el presente artículo de revisión sintetiza la evidencia científica publicada en los últimos 3 años (periodo 2019-2021) acerca de las manifestaciones orales de la COVID-19.

## Abstract (en)

COVID-19 is a currently pandemic disease that worries professionals in all areas of the Health Sciences. Current literature indicates that certain components of the stomatognathic system (such as lips, oral mucosa, tongue, and tonsils) express ACE2 receptors, responsible for the binding of SARS-CoV-2 to the human cell. This expression has a big impact on organular compromise, turning the oral cavity not only into an important route of viral entry but also into a region with the possibility of various manifestations such as xerostomia, halitosis, chemosensory alteration, opportunistic coinfection, and mucosal lesions. The fact that mouth manifestations are reported in the early stages of this disease suggests a possible predictive nature, which would make them very useful for diagnostic referral. In this sense, it is important that the dental professional knows how to recognize this early presentation symptomatology, being able to presumptively detect patients with COVID-19 in whom a significant systemic commitment has not yet been consolidated. This would allow the referral to medical consultation for definitive diagnostic purposes and, if correct, prevent both transmission and the advancement of the disease in the patient. Based on this important work, this review article synthesizes the scientific evidence published in the last 3 years (2019-2021 period) about the oral manifestations of COVID-19.

## INTRODUCCIÓN

En 2019, una enfermedad infecciosa apareció en Wuhan (China), la cual fue catalogada como COVID-19 y es causada por el nuevo coronavirus SARS-CoV-2 ^1^. Este virus con tropismo humano infecta generalmente el tracto respiratorio y genera síntomas como fiebre, tos seca, cefalea, entre otros ^2^.

El SARS-CoV-2 presenta 4 proteínas estructurales a nivel de superficie, entre las que destaca la proteína Spike (S) que, al unirse a un receptor diana (ACE2) presente en la célula huésped, permite el ingreso viral, tras lo que se producen la replicación y la propagación viral ^2^. La proteína S presenta 2 subunidades: S1, que se encuentra a nivel del dominio RBD y sirve para la unión del virus con el receptor ACE2; y S2, que genera la fusión de membranas entre la célula diana y el virus ^3^. Además, tanto el ingreso como la liberación de material genético viral se ven facilitados por la presencia de la proteasa TMPRSS2 en el tejido endotelial ^1^.

Con relación al ámbito odontológico, tanto la mucosa oral como la lengua y las glándulas salivales expresan ampliamente los receptores ACE2, lo que aumenta su vulnerabilidad a daño tisular ^4^. La afectación de la cavidad oral por la diseminación viral y consecuente tormenta de citocinas que origina una fuerte respuesta inflamatoria se ve reflejado en diferentes lesiones de mucosa, alteraciones quimiosensoriales como la disgeusia, desarrollo de xerostomía, aumento del riesgo de candidiasis oral y otros ^(1, 5)^. Una vez comprometida la cavidad oral, se debe tener en cuenta que la infección puede alcanzar las glándulas salivales mayores y convertir la saliva en un reservorio con potencial de propagación hacia otros órganos.

Al ser así, es fundamental que el profesional de la salud, en especial el odontólogo, pueda identificar las manifestaciones orales del paciente con COVID-19, aquellas que, según la literatura, se presentan en etapas tempranas del desarrollo de la enfermedad. Una clara identificación permitirá al odontólogo sospechar de casos COVID-19 positivos y, al reportarlo, prevenir futuras infecciones, facilitar el diagnóstico y dar un tratamiento precoz (tanto sistémico como oral), a fin de prevenir el agravio de la sintomatología y el compromiso sistémico que puede desarrollar el paciente ^1^^,^^6^. Es esta marcada serie de beneficios lo que hace necesario recopilar el conocimiento científico actual acerca de las manifestaciones orales de la COVID-19. 

La presente revisión narrativa ofrece una síntesis de la información disponible en la literatura científica actual acerca de las diferentes manifestaciones orales de la infección por SARS-CoV-2 y el consecuente padecimiento de COVID-19. 

## MATERIALES Y MÉTODOS

Se realizó una búsqueda no sistemática a fin de recolectar información publicada con relación a las manifestaciones orales de la COVID-19. La indagación de fuentes se realizó entre los meses de junio y agosto de 2021, e incluyó las bases de datos Science Direct, Medline y SciELO, así como los buscadores Scopus, PubMed y Google Académico. La temporalidad de las fuentes de información fue limitada a los últimos 3 años (periodo 2019-2021), con el fin de obtener información vigente respecto del tema. Las palabras o términos clave tomados en cuenta fueron los siguientes: “Infección por coronavirus”, “COVID-19”, “SARS-CoV-2”, “manifestaciones bucales” y “cavidad oral” (DeCS). Los términos en inglés fueron “Coronavirus infections”, “COVID-19”, “SARS-CoV-2”, “oral manifestations” y “oral cavity” (MeSH). La conjugación de términos priorizó la permanencia de “infección por coronavirus” y “manifestaciones bucales”, a fin centrar la información obtenida por los motores de búsqueda.

Posteriormente a la exploración de la literatura, la selección se inició con el análisis de títulos y resúmenes. Para ello, se tuvo en cuenta que dichas fuentes de información cubrieran alguno de los siguientes aspectos: receptores ACE2 e ingreso del virus; disfunción quimiosensorial; xerostomía, halitosis y candidiasis; lesiones de mucosa, periodonto, lengua y labios. También se tomó en cuenta que los artículos aborden la importancia del rol odontológico en la detección sintomatológica. Luego, se realizó la lectura de todos los documentos y se descartó aquellos no pertinentes para la presente revisión narrativa.

## RESULTADOS Y DISCUSIÓN

### Receptores ACE2, ingreso viral e inflamación

La infección y propagación mundial del SARS-CoV-2 se vio reflejada en la actual pandemia de COVID-19. Al ingresar el virus al organismo de un individuo, se desatan mecanismos de unión y replicación con el fin de infectar a los órganos que posean su receptor diana (ACE2). La infección deriva en signos identificados frecuentemente en los pacientes COVID-19 positivos: fiebre, tos, disnea, mialgia y fatiga ^1^. 

Además, la presencia de receptores ACE2 en células epiteliales de las glándulas salivales y mucosa bucal -mayormente en el dorso de lengua- ha condicionado la aparición de sintomatología relacionada, lo que influye en la propia patogenia de esta enfermedad ^2^. La proximidad de estos receptores celulares a la proteína S del virus genera una mayor facilidad de la propagación viral y, con esto, una mayor progresión de la COVID-19 a nivel poblacional ^3^.

En la cavidad oral, la expresión del receptor ACE2 se concentra en los estratos basal, espinoso y queratinizado de las células epiteliales, y se expresa más en la lengua que en el resto de la mucosa bucal ^2^. La actividad mucotrópica del SARS-CoV-2 y su desenlace en una tormenta de citocinas inducen a la inflamación en mucosa, lo que propicia la aparición de signos y síntomas de la infección en boca. Además, la evidencia científica reciente sugiere que la expresión específica de los receptores ACE2 y las proteasas TMPRSS2 en células gustativas de las papilas fungiformes y glándulas salivales submandibulares, parótidas y menores es un posible mecanismo patológico de la disfunción gustativa y xerostomía que han manifestado tempranamente a los pacientes con COVID-19 ^1^. 

### Alteración quimiosensorial: disgeusia y anosmia

Entre las manifestaciones orales por la COVID-19 que cursan con un inicio temprano y presentan altas prevalencias, se encuentran las alteraciones quimiosensoriales: disgeusia y anosmia ^7^^,^^8^. Estudios en pacientes hospitalizados indican la gran importancia de estos síntomas, particularmente en etapas tempranas de la enfermedad, y establecen su detección oportuna como una importante herramienta que lleve al diagnóstico y tratamiento efectivo de la infección por SARS-CoV-2, con miras en la reducción de su morbilidad y mortalidad ^9^^,^^10^.

Al ser la disgeusia -o alteración del gusto- aquella manifestación que ha llegado a presentarse con frecuencias de hasta el 88,8% en estudios multicéntricos ^11^, es vista como síntoma patognomónico de la infección temprana por SARS-CoV-2 ^12^. Además, pese a la falta de esclarecimiento acerca de la fisiopatología por la que cursa en relación a este virus, se han planteado varias posibles explicaciones al respecto, todas relacionadas con la distribución de los receptores ACE2 en los tejidos afectados.

Por su parte, la ruta fisiopatológica que sigue la anosmia -o pérdida del olfato- con relación a la COVID-19 no parece discrepar demasiado respecto de la observada en otras infecciones virales, y se da como resultado de alteraciones secundarias en el neuroepitelio olfatorio a causa del gran proceso inflamatorio que conlleva la infección ^13^. Estudios imagenológicos realizados en pacientes con anosmia persistente posinfección por SARS-CoV-2 sugieren que el inicio de estas alteraciones toman lugar en la hendidura olfatoria, lo que puede generar lesiones en las vías neuronales olfativas y relacionarse con la degeneración del bulbo olfatorio ^14^.

### Xerostomía, halitosis y candidiasis

La xerostomía, comúnmente llamada “boca seca”, es producto de una alteración en el funcionamiento normal de las glándulas salivales. Esta afección puede ocurrir debido a que el epitelio glandular salival también expresa los receptores ACE2, que son vulnerables a la infección por SARS-CoV-2 ^15^. En ocasiones, la xerostomía se produce como consecuencia directa de la COVID-19 y puede presentarse antes que otros síntomas considerados comunes, lo que facilita un diagnóstico temprano ^16^. Además de la xerostomía como efecto directo de la infección, una vez presentada, podría verse agravada por efectos secundarios a los medicamentos utilizados para tratar la sintomatología producida por la COVID-19, o también debido al uso continuo de la mascarilla ^17^^-^^19^.

Por otro lado, también se ve implicada la halitosis, definida como el mal olor procedente de la boca, que puede deberse a una mala higiene oral, a enfermedades de la cavidad oral o a causas extraorales. La aparición de la halitosis está comúnmente relacionada con afecciones intraorales, entre las que destaca la xerostomía, relación que se presenta con mayor frecuencia en pacientes ancianos con compromiso sistémico y consumo habitual de medicamentos que propician su aparición ^20^. Al igual que la xerostomía, la halitosis puede producirse como un efecto indirecto de la COVID-19, ello debido a la respiración bucal encausada en el uso de la mascarilla. Además, se ha encontrado asociación entre la halitosis y las coinfecciones derivadas del SARS-CoV-2, ya que estas pueden tener una modulación del ambiente oral a favor de la proliferación de la microbiota asociada con la halitosis. Un ejemplo de esto es el reporte de halitosis grave asociada a gingivitis necrotizante, lo cual sugiere el impacto que pueden tener las coinfecciones relacionadas con la COVID-19 ^19^.

Otra manifestación oral de la COVID-19 que guarda relación con la xerostomía es la candidiasis oral. Esta se ha registrado en algunos pacientes con COVID-19 gravemente afectados, especialmente en aquellos con enfermedad predisponente o ingesta de antibióticos ^5^. Además, las dosis altas de corticosteroides usados frente a la crisis inflamatoria generada por la COVID-19 también podrían precipitar la aparición de esta enfermedad ^15^.

### Lesiones de mucosa y periodonto

La mucosa oral es una barrera constituida de epitelio escamoso y corion, que desempeña funciones de revestimiento y protección en su porción masticatoria, y de percepción de sensaciones y secreción en su porción especializada ^21^^,^^22^. En odontología, son comunes las infecciones virales orales que se asocian con lesiones en la mucosa. Sabemos que la actual COVID-19 afecta la mucosa bucal, debido a su alto contenido de receptores afines con el virus, principalmente en el estrato queratinizado. Por tanto, existen altas probabilidades de encontrar lesiones asociadas con el padecimiento de esta enfermedad en la cavidad oral ^21^. 

La afinidad del SARS-CoV-2 por las células epiteliales de la cavidad oral aumenta el riesgo de infección. Una vez que el individuo es contagiado, se compromete el equilibrio de su microbiota; luego se presentan alteraciones en la mucosa, glándulas salivales y tejidos gingivales ^23^^,^^24^. Además, el compromiso inmunológico que conlleva la corticoterapia, actualmente indicada para tratar la sintomatología de la COVID-19, sumada a la limitada higiene oral de los pacientes, expone a una coinfección tanto viral como bacteriana ^25^^,^^26^.

En la literatura, múltiples reportes de casos confirman la presencia de manifestaciones orales en pacientes COVID-19 positivos y sospechosos. Entre estas, destacan úlceras, queilitis erosivas, angina bullosa hemorrágica, máculas, placas, erosiones, costras -con o sin hemorragia-, petequias, eritemas y pápulas no sangrantes; todas estas repartidas en el paladar duro, el blando, la gingiva y los carrillos, y que son encontradas durante el examen intraoral al paciente o de forma remota mediante fotografías ^24^^,^^26^^-^^30^. El tipo de lesión más frecuentemente detectado en la infección por SARS-CoV-2 es la ulcerativa. Se cree que cuando el virus y el receptor ACE2 interaccionan se produce una alteración en el revestimiento epitelial, lo que aumenta la permeabilidad de los queratinocitos y permite un mayor y más fácil ingreso de patógenos que, luego de replicarse, generan la ulceración del tejido ^24^.

Es importante mencionar que, desde el punto de vista histológico, luego de realizar biopsias a este tipo de lesiones, se observaron alteraciones a nivel epitelial y del corion. En estas se ha podido notar la vacuolización de queratinocitos en el estrato espinoso, células de inflamación crónica en la lámina propia, además de hemorragias y congestiones vasculares en el tejido subepitelial ^31^.

### Lesiones de lengua y labios

La existencia de receptores ACE2 en las papilas gustativas y el dorso de lengua ha propiciado un aumento de la actividad viral en su mucosa. Se ha reportado la aparición de aftas dolorosas en el borde lateral de la lengua, sumada a múltiples úlceras amarillentas y puntiformes en el dorso de esta, asemejándose a la etapa tardía de la enfermedad herpética ^30^^,^^32^. Asimismo, se han reportado cambios en la sensibilidad de la lengua en pacientes que sufrieron la enfermedad de la COVID-19, presentándose placa blanca persistente en el dorso e hinchazón en paladar y encías ^22^.

Otra de las zonas intraorales más comúnmente afectadas por la COVID-19, además del dorso lingual es la mucosa labial, específicamente del labio inferior ^33^. Se pueden presentar erosiones, ulceraciones o áreas necróticas con desenlace en costras hemorrágicas de la zona cutánea ^34^, placas erosivas, hinchazón, enrojecimiento y agrietamiento que, según la gravedad, pueden ocasionar dolor y afectar tanto la masticación como el habla ^35^^,^^36^. 

### Origen indirecto de las manifestaciones orales

Entre los factores que contribuyen al padecimiento de lesiones primarias y secundarias en mucosa y periodonto de forma indirecta encontramos la farmacoterapia durante el periodo de tratamiento contra la COVID-19, una mala higiene en el tiempo de hospitalización y la fatiga o estrés al cual es sometido el paciente, lo cual puede reactivar enfermedades como el herpes ^29^^-^^31^. 

Por otro lado, se debe mencionar que las manifestaciones orales de la COVID-19 también se han atribuido a efectos adversos de los tratamientos, al uso de sustancias antisépticas aplicadas por vía oral, a la higiene bucal deficiente, a lesiones traumáticas e inmunosupresión asociada con fármacos, lo cual predispone al paciente a infecciones oportunistas y, por consiguiente, a un deterioro de su salud sistémica ^28^. Un ejemplo de esto es la interacción virus-bacteria, la cual puede conducir a modificaciones en las citocinas y respuestas linfocíticas que provocan la exacerbación de la enfermedad pulmonar en el paciente ^37^. Con respecto a los tipos de lesiones cutáneas encontradas y que podrían comprometer la región perioral, la mayoría fue de tipo exantema u otras lesiones inflamatorias, entre las cuales predominaron las máculas eritematosas, seguidas de las lesiones vasculíticas del tipo úlceras y, por último, las lesiones que podrían estar relacionadas a reacciones de hipersensibilidad a fármacos ^38^.

## CONCLUSIONES

Es necesario poner atención a la cavidad oral como centro inicial de la infección por SARS-CoV-2, debido a la alta expresión de receptores ACE2 en mucosa bucal que permiten la entrada y posterior propagación viral. Esta propagación puede producir alteraciones quimiosensoriales como la anosmia y la disgeusia, que son de gran ayuda para el diagnóstico presuntivo de la COVID-19. También puede existir afectación de glándulas salivales, y es común que se manifieste como xerostomía y la consiguiente aparición de halitosis. Además, la infección por SARS-CoV-2 aumenta el riesgo de coinfección oportunista, lo que puede causar candidiasis oral por predisposición farmacológica.

Las lesiones en mucosa bucal y periodonto más frecuentemente reportadas en pacientes COVID-19 positivos fueron las queilitis erosivas, anginas bullosas, máculas, placas, erosiones, costras, petequias, eritemas, pápulas y úlceras (mayor prevalencia y agravamiento con la edad). Las manifestaciones más frecuentes en la zona lingual fueron aftas en bordes laterales, placas blancas en dorso y reactivación de lesiones herpéticas acompañadas de úlceras amarillentas. Además, se debe tener en cuenta que la aparición de lesiones bucales asociadas con la COVID-19 pueden presentarse de forma directa o indirecta, y destacan en el último caso la farmacoterapia, la inmunosupresión, la mala higiene y el estrés.

## Financiamiento:

Autofinanciado
